# Volatile and Non-Volatile Organic Compounds Stimulate Oviposition by Aphidophagous Predators

**DOI:** 10.3390/insects11100683

**Published:** 2020-10-10

**Authors:** Eric W. Riddick

**Affiliations:** Agricultural Research Service, USDA, Stoneville, MS 38776, USA; eric.riddick@usda.gov

**Keywords:** chemical ecology, natural enemies, natural products, reproduction

## Abstract

**Simple Summary:**

Evidence that volatile and non-volatile organic compounds stimulate aphid-eating predators to lay eggs is scattered throughout the scientific literature. The objectives of this review are to compile records indicating that organic compounds stimulate egg laying, calculate an egg production ratio for stimulated predators, and determine if the egg production ratio is correlated with vapor pressure and molecular weight of active compounds. Results indicated that both volatile and non-volatile compounds stimulated egg laying behavior by coccinellid beetles and syrphid flies, but not chrysopid lacewings. The egg production ratio was greatest for syrphid flies. Regardless of predator taxa, the egg production ratio was negatively and positively correlated with molecular weight and vapor pressure, respectively. In conclusion, volatile organic compounds stimulate syrphid flies to lay more eggs than coccinellid beetles and chrysopid lacewings. Volatile organic compounds could be used to manipulate predators to lay more eggs in mass rearing systems or on aphid-infested plants in greenhouses or high tunnels.

**Abstract:**

Introduction: Evidence that volatile organic compounds (VOCs) and non-VOCs stimulate oviposition by aphidophagous predators is scattered throughout the literature. The objectives of this review are to (1) compile records indicating that VOCs and non-VOCs are responsible for oviposition stimulation, (2) calculate an egg production ratio (EPR) for stimulated predators, and (3) determine if EPR is correlated with vapor pressure and molecular weight of active compounds. Methods: The USDA (United States Department of Agriculture), National Agricultural Library, online digital catalog system was used to retrieve abstracts, then the full text of manuscripts on oviposition stimulants for predators. Oviposition-stimulating VOCs and non-VOCS were tabulated with molecular weights and vapor pressure estimates. EPRs were calculated for stimulated coccinellids, syrphids, and chrysopids. Results: Both VOCs and non-VOCs stimulated oviposition behavior by coccinellids and syrphids, but not chrysopids. EPR was greatest for syrphids. Two VOCs, (E)-β-farnesene and 3-methyl-2-butenal, stimulated very high EPR values by the syrphid *Episyrphus* *balteatus*. Regardless of predator taxa, EPR was negatively and positively correlated with molecular weight and vapor pressure, respectively. Conclusions: Syrphids (rather than coccinellids or chrysopids) produce more eggs in response to VOCs. Organic compounds with low-to-moderate molecular weights and moderate-to-high vapor pressures might be most effective oviposition stimulants for aphidophagous predators.

## 1. Introduction

Evidence that plants produce volatile organic compounds (VOCs) to deter insect herbivores and communicate between plants (or between parts of the same plant) has accumulated over the last several decades [1,2,3,4,5]. VOCs are also known to attract predators and parasitoids to herbivore (aphid)-infested plants [6,7,8,9,10,11,12]. Less well known is the capacity of VOCs as well as non-VOCs to affect oviposition behavior in aphid predators and hardly anything is known on VOCs or non-VOCs functioning as oviposition stimulants for hymenopterous aphid parasitoids [13,14]. Hence, this review focuses on reviewing evidence of oviposition stimulatory compounds for aphid predators, i.e., coccinellids (Coleoptera: Coccinellidae), syrphids (Diptera: Syrphidae), and chrysopids (Neuroptera: Chrysopidae). 

The USDA (United States Department of Agriculture), National Agricultural Library, online digital catalog system (DigiTop) Navigator platform, which includes scientific research databases (such as Web of Science, CAB Abstracts, BIOSIS Previews, Zoological Record, Scopus, AGRICOLA, etc.) was used to retrieve abstracts, then the full text of manuscripts. The key words “predators and oviposition stimulant(s)” or “oviposition stimulants” were used to begin the initial search for relevant manuscripts focusing directly (or indirectly) on organic compounds as stimulants for oviposition or organic compounds to increase egg production. 

In the retrieved manuscripts, VOCs and non-VOCs found to stimulate oviposition were tabulated, with minimal effective concentrations, for each species. VOCs were characterized as having a vapor pressure of at least 0.001 mm Hg (25 °C). Vapor pressure (a measure of volatility), molecular weight (g/mol), and other physical characteristics of these compounds were retrieved from several online reference sources. A Kruskal–Wallis analysis of variance (K-W ANOVA) was used to compare the egg production ratios (EPRs) amongst coccinellids, syrphids, and chrysopids. EPR was the ratio of the mean number of eggs (or egg clutches) in test/control arenas after exposure to VOCs or non-VOCs. A Spearman rank correlation analysis tested if vapor pressure and molecular weight were correlated with EPR. The objectives of this review are to (1) compile records indicating that VOCs and non-VOCs are responsible for oviposition stimulation, (2) calculate an egg production ratio (EPR) for stimulated predators, and (3) determine if EPR is correlated with vapor pressure and molecular weight of active compounds.

## 2. Evidence of Oviposition Stimulation 

### 2.1. Tabulated Data on Oviposition Stimulation by VOCs and Non-VOCS 

To simplify the ensuing review of the role of VOCs and non-VOCs in oviposition stimulation by aphidophagous predators, two tables were created. Table 1 provides data on VOCs and non-VOCs stimulating oviposition behavior in coccinellids, syrphids, and chrysopids, with minimal effective concentrations. The egg production ratio (EPR*) for each representative predator is also reflected in the table. Table 2 indicated the VOCs and non-VOCs with accompanying molecular weight, vapor pressure, and odor strength characteristics, regardless of predator taxa. In this review, VOCs had a vapor pressure higher than 0.001 mm Hg, 25 °C, while that of non-VOCs was lower than 0.001 (Table 2). 

### 2.2. Oviposition Stimulants and Coccinellids

The ladybird beetles (coccinellids) are well-known natural enemies of aphids throughout the world [31]. Although prey (e.g., aphids), prey products (e.g., honeydew), or plants have been shown to serve as cues for oviposition in coccinellids [32], very few studies have reported that plant or prey-derived VOCs or non-VOCs stimulate oviposition. In separate experiments, treatment of substrate surfaces with salicylic acid, *o*-coumaric acid, and protocatechuic acid stimulated oviposition by *Coleomegilla maculata lengi* Timberlake females more than the control, untreated surfaces [15]. Out of nine chemical compounds formulated mostly in water, guaiacol and resorcinol were the most effective oviposition stimulants for *C*. *maculata lengi* [16]. 

*Coleomegilla maculata* (DeGeer) females were occasionally stimulated to oviposit in small bioassay cages in the laboratory, when exposed to taxifolin, quercetin, and naringenin [17]. In separate 250 mL lab cage bioassays, synthetic quercetin and taxifolin caused females to oviposit at the base of cages rather than on cage walls or lids, which were preferred oviposition sites in the absence of the compounds and any oviposition substrates. In a follow-up study, quercetin altered *C*. *maculata* oviposition behavior in 1 L lab cages, housing 10 females, even in the presence of a preferred oviposition substrate, such as white tissue paper [18]. Lastly, a final study tested the effectiveness of 2,4-dihydroxybenzoic acid (DHBA), which is a degradation product of the flavonoid morin, to stimulate oviposition by *C*. *maculata* [33]. Although DHBA altered the oviposition site selection behavior of *C*. *maculata* in replicate 1 L cages housing 10 females, it did not cause females to lay more egg clutches or generate more eggs per clutch. Another interesting result was that females had to physically contact, i.e., touch or taste DHBA, to elicit any change in oviposition site selection in replicate 500 mL cages. This suggests that tactile and gustatory receptors are more important than olfactory receptors in perception of DHBA and perhaps other high molecular weight compounds. DHBA was considered a weak oviposition stimulant [33].

In laboratory experiments, limonene and β-caryophyllene altered prey location and oviposition behavior of the ladybird beetle *Harmonia axyridis* (Pallas) [19]. When applied to rubber septa, then attached to the stems of potted broad bean (*Vicia faba* L.) plants, both chemicals increased *H*. *axyridis* oviposition on plants. Other test volatiles, i.e., (Z)-3-hexen-1-ol, β-pinene and (E)-β-farnesene, did not stimulate *H*. *axyridis* oviposition in this study. Note that (E)-β-farnesene is an aphid alarm pheromone [34]. When limonene or (E)-β-farnesene was applied to controlled-release dispensers, which were then attached to yellow water pan traps, in a 5 ha chicory (*Cichorium intybus* L.) field, *H*. *axyridis* adults were attracted to both compounds [19]. There are very few field-based studies to demonstrate that volatile chemicals are important attractants or oviposition stimulants [35]. More research is necessary to use chemical cues to manipulate ladybird beetle prey searching behavior [36] and encourage oviposition on plants infested with aphids under greenhouse or open field conditions. 

In summarizing this section, the discovery of high molecular weight, non-volatile organic compounds (non-VOCs) such as the flavonoids quercetin and taxifolin as oviposition stimulants for lab-reared *C*. *maculata* was novel, because this coccinellid had no prior exposure to either compound [17,18]. Are olfactory receptors important in detection of non-VOCs? Female coccinellids must touch or taste non-VOCs such as flavonoids and flavonoid derivatives before oviposition stimulation can occur [17,18,33], suggesting that olfactory receptors have a limited role in detection of non-VOCs and oviposition stimulation in coccinellids. Yet, one coccinellid species, *H*. *axyridis*, was stimulated to oviposit when arenas were treated with limonene and β-caryophyllene, both of which are considered VOCs. More research is necessary to understand the physiological mechanisms responsible for odor detection and oviposition stimulation in coccinellids.

The egg production ratio (EPR) of coccinellids stimulated by non-VOCs and VOCs in the section ranged from 1.3 to 6.0. An examination of the EPRs in Table 1 and vapor pressure and molecular weight values in Table 2 indicated that EPRs were sometimes lower for coccinellids exposed to high molecular weight non-VOCs rather than moderate-to-low molecular weight VOCs. As a case-in-point, *C*. *maculata* had an EPR of 1.3 after exposure to quercetin but had an EPR of 6.0 after exposure to resorcinol. Quercetin and resorcinol are characterized in this study as a non-VOC and VOC, respectively. Note that chemical concentrations used in bioassays listed beside organic compounds in Table 1 were too variable amongst the different studies to perform an analysis of the effects of VOC or non-VOC concentration on EPR of coccinellids.

### 2.3. Oviposition Stimulants and Syrphids

The hoverflies (syrphids) are widely known as contributing to the suppression of aphids in natural and managed ecosystems throughout the world. Only the larval stages are predatory; the adults are pollinators, since they visit flowers to obtain pollen for egg maturation and nectar for energy [37,38]. Syrphid females responded to the density and distribution of prey (aphids) and adjusted the rate of oviposition, accordingly; yet, this response was dependent on syrphid species [39]. In laboratory experiments using a flight cage, the oviposition behaviors of two syrphids, *Platycheirus albimanus* (F.) and *Episyrphus balteatus* (DeGeer), were determined in response to aphid honeydew, artificially applied to ears of wheat [40]. Honeydew caused an ovipositional response in *E*. *balteatus* but not *P*. *albimanus*. Honeydew from the aphids *Metopolophium dirhodum* Walker and *Acyrthosiphon pisum* Harris stimulated oviposition; honeydew from *Microlophium carnosum* (Bukt.) did not. Increasing the honeydew concentration stimulated an increase in the number of eggs laid by *E*. *balteatus* [40].

The syrphid *Metasyrphus corollae* F. was a predator of the bean aphid *Aphis fabae* Scopoli. In laboratory experiments, *M*. *corollae* oviposited more eggs on broad bean (*V. faba*) leaves treated with a crude extract of its prey, *A*. *fabae*, in comparison to control [41]. In addition, the ovipositional response was concentration dependent; more eggs were laid as the concentration of the crude extract increased. The crude extract, representative of 100 aphid individuals, homogenized in 1 mL distilled water elicited *M*. *corollae* females to lay the highest number of eggs in laboratory bioassays [41].

Active fractions of aphid (*Aphis fabae* Scopoli) extracts revealed the presence of straight chain hydrocarbons, ranging from docosane (C_22_H_46_) to octacosane (C_28_H_58_), based on gas chromatography-mass spectrometry analysis [20]. Laboratory bioassays using live *M*. *corollae* females indicated that tricosane (C_23_H_48_) was the most active compound regarding oviposition stimulation. In addition, a mixture of hydrocarbons, including tricosane, tetracosane (C_24_H_50_), pentacosane (C_25_H_52_), hexacosane (C_26_H_54_), and octacosane (C_28_H_58_) stimulated more *M*. *corollae* oviposition than any other mixture or tricosane alone [20]. Mechanoreceptors on the antennae of *M*. *corollae* females could be involved in reception of hydrocarbons released from pore canals in the cuticle or from cornicle secretions of *A*. *fabae* females [20].

In a similar study, prior exposure to aphid (*A*. *fabae*) extract or hexacosane, and the mixture of the two, caused *M*. *corollae* females to lay more eggs than those without previous exposure, in petri-dish bioassays [21]. Aphid extract and hexacosane also increased *M*. *corollae* searching behavior, i.e., length of search path and number of turns along the path. 

In laboratory experiments, green leaf volatiles (*Z*)-3-hexenol and (E)-β-farnesene induced the syrphid *Episyrphus balteatus* (De Geer) to oviposit on aphid-free bean plants [22]. Note that (E)-β-farnesene attracted syrphids (as well as other predators) in previous research in laboratory and field experiments [42]. *Episyrphus balteatus* and other syrphids rely on chemical cues from green leaf volatiles and aphid pheromones to select suitable oviposition sites [22]. Chemical cues from green leaf volatiles and aphid alarm pheromone were important in oviposition site selection by predators. Other factors including habitat, host plant, aphid species, aphid availability, presence of competitors (intra- and interspecific), and female age were also influential [37]. 

An experimental device was created to encourage oviposition and predation of aphids by *E*. *balteatus* [23]. The device consisted of a plastic lamella, i.e., oviposition substrate, treated with (E)-β-farnesene and/or concentrated sugars, i.e., 10–30% sucrose, fructose, or glucose. Females laid more eggs in the presence of (E)-β-farnesene on rubber septa than any combination of this compound with sugars or control. Placement of the plastic lamella with 15 *E*. *balteatus* eggs on plants infested with aphids (*A*. *fabae* and *A. pisum*) resulted in larval predation of 500 aphids within 10 days [23]. 

In a related study, a bacterium discovered in *A*. *pisum* honeydew was shown to function as an ovipositional stimulant for *E*. *balteatus* [24]. The bacterium was identified as *Staphylococcus sciuri* Koos (Bacilles: Staphylococcaceae). Chemical compounds produced by *S*. *sciuri* were confirmed as the source of ovipositional stimulants. The compounds were 3-methyl-2-butenal and 2-methylbutanoic acid [24]. The discovery that bacteria can produce compounds capable of stimulating oviposition in *E*. *balteatus* warrants further investigation.

In summarizing this section, syrphid females depend on chemical cues from aphids and aphid honeydew to stimulate oviposition in the laboratory and field. Non-VOCs (e.g., tricosane) and VOCs (e.g., (E)-β-farnesene) functioned as oviposition stimulants for *E*. *balteatus*. Tricosane has a relatively high molecular weight and low vapor pressure; (E)-β-farnesene has a moderate molecular weight and moderate vapor pressure (Table 2). EPRs for syrphids ranged from 2.1 to 18.0 after exposure to tricosane and (E)-β-farnesene, respectively (Table 1). As in the section on coccinellids, chemical concentrations listed beside organic compounds in Table 1 were too variable amongst the different studies to conduct any meaningful analysis of the effects of VOC or non-VOC concentration on EPR of syrphids. 

### 2.4. Oviposition Stimulants and Chrysopids

*Chrysoperla carnea* (Stephens), known as the common green lacewing, but now recognized as a complex of sibling species, has been an important predator of small soft-bodied plant pests (e.g., aphids) in agroecosystems throughout the world [43]. Adults utilized pollen and insect secretions (honeydew) for maintenance and reproduction, i.e., oviposition [44]. In previous research, *Chrysoperla carnea* adults were attracted to tryptophan in artificial honeydews [45]. Experiments in the laboratory and field were designed to discover effective ovipositional attractants/stimulants for *C. carnea* to enhance its predation capacity in cropping systems [25]. Acid hydrolyzed L-tryptophan (15 d old) was found to attract male and female *C*. *carnea* in the laboratory. When sprayed on filter paper at a concentration of 33.3 mg/mL [46], 15 d old L-tryptophan stimulated female *C*. *carnea* to lay the highest number of eggs, in comparison to filter paper treated with pure commercial honey or control. In the field, results were not as promising; only 1 d old L-tryptophan induced *C*. *carnea* oviposition on cotton plants [25]. 

Applying acid hydrolyzed 1 d old L-tryptophan [46] to cotton foliage resulted in more *C*. *carnea* eggs (progeny) in treated than in control plots in 2003, but not in 2002 [26]. Significantly fewer aphids (species not indicated) were present in treated than control plots in 2002, but not in 2003.

In a field study, a chemical trap/lure consisting of a mixture of acetic acid, methyl salicylate, and phenylacetaldehyde in 1:1:1 ratio, attracted *C*. *carnea* females in organic apricot and peach orchards [27]. Methyl salicylate (MeSA) is a common herbivore-induced plant volatile, which attracts insect predators to aphid-infested plants [11,47,48]. Phenylacetaldehyde was an attractant for *C*. *carnea* [49,50]. The chemical lure caused a higher degree of oviposition by *C*. *carnea* females than the control trap in one of two consecutive field seasons [27]. In another field experiment, the chemical lure (1:1:1 mixture of acetic acid, methyl salicylate, and phenylacetaldehyde) significantly increased the number of *C*. *carnea* eggs laid on young cherry trees in an orchard [28]. More eggs were found in the vicinity of the lure, which was positioned in the center of the trees, than in other locations.

Experiments in a greenhouse using artificial plants inside replicate cages indicated that *Chrysoperla rufilabris* (Burmeister) females were attracted and showed a tendency to lay more eggs in the vicinity of rubber septa baited with MeSA than control [29]. Yet, significantly more eggs were only laid on artificial plants baited with MeSA in comparison to control. 

In behavioral bioassays in the laboratory, *Chrysopa phyllochroma* Waesmael females were significantly attracted to three plant volatiles, linalool, (Z)-3-hexenyl acetate, and (3E)-4,8-dimethyl-1,3,7-nonatriene [30]. When 5 μL of each compound was placed in an upper corner of replicate 100 L cages, all three compounds significantly increased *C*. *phyllochroma* oviposition within 10 cm of the compounds, in comparison to control. These observations suggest that the three plant volatiles functioned as oviposition stimulants.

In summarizing this section, chrysopid females responded to several VOCs in laboratory bioassays and the field. A mixture of three VOCs (acetic acid, methyl salicylate, and phenylacetaldehyde) stimulated oviposition by *C*. *carnea* in the field. The molecular weights of the independent compounds were low-to-moderate; vapor pressures were moderate-to-high (Table 2). EPRs for chrysopids ranged from 2.3 to 12.0 for L-tryptophan and the mixture of acetic acid, methyl salicylate, and phenylacetaldehyde, respectively (Table 1). The effect of VOC or non-VOC concentration on EPR of chrysopids was not determined.

## 3. Synthesis

The demarcation between VOCs and non-VOCs has not been clearly defined in the literature. For this study, any compound with a vapor pressure greater than 0.001 mm Hg at 25 °C was considered a VOC; less than 0.001 mm Hg was considered a non-VOC (Table 2). When comparing the EPR amongst predator groups, coccinellids, syrphids, and chrysopids, significantly more eggs were produced by syrphids than coccinellids, but not chrysopids (*H* = 8.69; df = 2; *p* = 0.013; *n* = 27 measurements). In addition, EPR did not differ significantly between coccinellids and chrysopids. The median EPR values (with 25 and 75% C.I. (confidence intervals) were 2.20 (1.5, 3.1) for coccinellids, 5.75 (3.6, 10.9) for syrphids, and 2.60 (2.2, 3.4) for chrysopids. Sample size was 11, 8, and 8 EPR measurements for coccinellids, syrphids, and chrysopids, respectively.

EPRs were compared with molecular weight and vapor pressure (if available) of associated VOCs and non-VOCs in Table 2. The pooled data (because of limited sample size and lack of vapor pressure for some compounds and mixtures) indicated that EPR and molecular weight were negatively correlated; EPR decreased as molecular weight increased (*r* = −0.547, *p* = 0.0058; *n* = 24). EPR and vapor pressure were positively correlated; EPR slightly increased as vapor pressure increased (*r* = 0.457, *p* = 0.0478; *n* = 19). Vapor pressure and molecular weight were highly correlated; vapor pressure decreased as molecular weight increased (*r* = −0.820, *p* < 0.0001; *n* = 19). In summarizing this section, non-VOCs were heavier molecules that typically did not yield high EPR values in comparison to VOCs. Based on the correlation analyses, molecular weight and vapor pressure are important parameters in predicting EPRs. 

The effects of VOC volatility, i.e., vapor pressure on odor detection and behavior modification by natural enemies are poorly known [51]. Olfactory receptors on antennae or mouthparts of predators and parasitoids are involved in the detection of VOCs [6,22,52]. In this review, female syrphids and chrysopids must detect VOCs with olfactory receptors, because of the volatile nature of these organic compounds. VOCs can have a moderate-to-high odor strength (see Table 2), but low rate of persistence on treated substrates or plant foliage. In contrast, mechanoreceptors and/or gustatory receptors could be more important than olfactory receptors in detection of non-VOCs by coccinellids [33].

## 4. Conclusions

This review compiled information on the utilization of oviposition stimulating compounds by aphidophagous predators, i.e., coccinellids, syrphids, and chrysopids. VOCs and non-VOCs functioned as stimulants. Because of their volatility, VOCs can be detected via olfactory chemoreceptors located on the mouthparts and/or antennae of coccinellids, syrphids, and chrysopids. In contrast, non-VOCs can be detected by contact mechanoreceptors and taste (gustatory) receptors on mouthparts of coccinellids. Compounds with the highest molecular weights are typically solids and non-volatile and have low-to-no detectable vapor pressure. 

Based on correlation analysis of pooled data, vapor pressure decreased as molecular weight increased. EPR decreased as molecular weight increased, and EPR slightly increased as vapor pressure increased.

A detailed knowledge of the vapor pressure could provide clues to successful deployment of VOCs and non-VOCs as oviposition stimulants for aphidophagous predators in commercial rearing operations and in the field (greenhouses or high tunnels). Compounds with high vapor pressures would be odoriferous and could be used to pull predators to artificial substrates or infested plants, from a distance. On the other hand, compounds with low vapor pressures would only pull predators to the substrate via direct contact. A strategy of deploying both compound types would be more effective than just using one or the other type, because the more volatile (high vapor pressure) compounds will dissipate in short time, while the less volatile (low vapor pressure) compounds will persist on the substrate for a longer period. More research is needed to advance the commercial utilization of VOCs and non-VOCS to improve mass production and deployment of aphidophagous predators for augmentative biological control. 

## Figures and Tables

**Table 1:** Volatile and non-volatile organic compounds (VOCs) stimulating oviposition behavior in coccinellids, syrphids, and chrysopids, with minimal effective conc., and egg production ratio (EPR*).

Predator	Compound Name (Concn)	EPR*	Reference
*Coleomegilla maculata lengi* (Col.: Coccinellidae)	salicylic acid (0.04 mg/cm^2^)	3.1	[15]
	*o*-coumaric acid (0.04 mg/cm^2^) protocatechuic	2.2	
	acid (0.04 mg/cm^2^)	2.6	
	guaiacol (1%)	5.0	[16]
	resorcinol (0.2%)	6.0	
*Coleomegilla maculata*	quercetin (0.004 mg/mL)	1.5	[17]
	taxifolin (0.004 mg/mL)	1.4	
	naringenin (0.004 mg/mL)	1.5	
	quercetin (0.008 mg/mL)	1.3	[18]
*Harmonia axyridis* (Col: Coccinellidae)	limonene (0.10 μg/μL)	2.2	[19]
	β-caryophyllene (0.10 μg/μL)	2.0	
*Metasyrphus corollae* (Dip.: Syrphidae)	tricosane (0.5 mg/mL)	2.1	[20]
	tricosane + tetracosane + pentacosane +	6.8	
	hexacosane + octacosane mix (2.5 mg/mL)		
	hexacosane (0.5 mg/mL)	4.7	[21]
*Episyrphus balteatus* (Dip.: Syrphidae)	(Z)-3-hexenol (0.40 μg/μL)	8.5	[22]
	(E)-β-farnesene (0.40 μg/μL)	18.0	
*E.* *balteatus*	(E)-β-farnesene (0.40 μg/μL)	3.6	[23]
	3-methyl-2-butenal (0.35 ng/μL)	11.8	[24]
	2-methyl-butanoic acid (0.024 ng/ μL)	3.6	
*Chrysoperla carnea* (Neu: Chrysopidae)	L-tryptophan (33.3 mg/mL)	2.3	[25,26]
	L-tryptophan (33.3 mg/mL)-field	2.8	
*C. carnea*	acetic acid + methyl salicylate +	12.0	[27,28]
	phenylacetaldehyde mixture (300 mg)-field.	3.4	
*Chrysoperla rufilabris* (Neu: Chrysopidae)	methyl salicylate (1.0 mg/mL)-greenhouse	2.1	[29]
*Chrysopa phyllochroma* (Neu: Chrysopidae)	linalool (5 μL)	3.3	[30]
	(Z)-3-hexenyl acetate (5 μL)	2.2	
	(3E)-4,8-dimethyl-1,3,7-nonatriene (5 μL).	2.4	

* EPR; the average number of eggs or egg clutches produced in test/control treatments.

**Table 2:** List of VOCs and non-VOCs mentioned in this study with molecular weight, vapor pressure, and odor strength characteristics, regardless of predator taxa. In this review, VOCs^†^ were characterized as having a vapor pressure greater than 0.001 mm Hg, 25 °C.

Compound	Molecular Formula	Physical State	Molecular Weight (g/mol)	Vapor Pressure (mm Hg, 25°C)	Odor Strength	Web. *
*o*-coumaric acid	C_9_H_8_O_3_	crystalline powder	164.2	1.9 × 10^−5^	n/a	i, ii
resorcinol^†^	C_6_H_6_O_2_	solid	110.1	0.002	medium	ii
salicylic acid^†^	C_7_H_6_O_3_	powder	138.1	1.0	low	ii
guaiacol^†^	C_7_H_8_O_2_	liquid or solid	124.1	0.18	high	ii
protocatechuic acid	C_7_H_6_O_4_	solid	154.1	n/a	low	ii
quercetin	C_15_H_10_O_7_	powder	302.2	2.8 × 10^−14^	n/a	i
taxifolin	C_15_H_12_O_7_	powder	304.2	1.3 × 10^−13^	n/a	iii
naringenin	C_15_H_12_O_5_	powder	272.2	n/a	n/a	ii
limonene^†^	C_10_H_16_	clear liquid	136.2	1.55	medium	ii
β-caryophyllene^†^	C_15_H_24_	oily liquid	204.3	0.01	medium	ii
(Z)-3-hexenol^†^	C_6_H_12_O	clear liquid	100.2	1.04	high	ii
(E)-β-farnesene^†^	C_15_H_24_	clear liquid	204.3	0.01	medium	ii
3-methyl-2-butenal^†^	C_5_H_8_O	clear liquid	84.1	8.25	high	ii
(Z)-3-hexenyl acetate^†^	C_8_H_14_O_2_	clear liquid	142.2	1.22	high	ii
2-methyl-butanoic acid^†^	C_5_H_10_O_2_	clear liquid	102.1	0.55	medium	ii
tricosane	C_23_H_48_	waxy solid	324.6	1.2 × 10^−5^	n/a	ii
tetracosane	C_24_H_50_	waxy solid	338.7	6.0 × 10^−6^	n/a	ii
pentacosane	C_25_H_52_	waxy solid	352.7	2.0 × 10^−6^	n/a	ii
hexacosane	C_26_H_54_	waxy solid	366.7	1.2 × 10^−5^	n/a	ii
octacosane	C_28_H_58_	waxy solid	394.8	n/a	n/a	ii
L-tryptophan	C_11_H_12_N_2_O_2_	crystalline powder	204.2	n/a	n/a	ii
acetic acid^†^	C_2_H_4_O_2_	clear liquid	60.0	15.7	high	ii
methyl salicylate^†^	C_8_H_8_O_3_	clear liquid	152.1	0.03	medium	ii
linalool^†^	C_10_H_18_O	clear liquid	154.2	0.02	medium	ii
phenylacetaldehyde^†^	C_8_H_8_O	oily liquid	120.1	0.37	high	ii
(3E)-4,8-dimethyl-1,3,7-nonatriene	C_11_H_18_	liquid	150.3	n/a	n/a	i

^†^ VOC. n/a; vapor pressure not available. * Websites: (i) https://pubchem.ncbi.nlm.nih.gov, (ii) http://www.thegoodscentscompany.com, (iii) https://www.chemspider.com.
